# Increased Serum Interleukin-9 Levels in Rheumatoid Arthritis and Systemic Lupus Erythematosus: Pathogenic Role or Just an Epiphenomenon?

**DOI:** 10.1155/2015/519638

**Published:** 2015-05-19

**Authors:** Andréa Tavares Dantas, Claudia Diniz Lopes Marques, Laurindo Ferreira da Rocha Junior, Mariana Brayner Cavalcanti, Sayonara Maria Calado Gonçalves, Pablo Ramon Gualberto Cardoso, Henrique de Ataide Mariz, Moacyr Jesus Barreto de Melo Rego, Angela Luzia Branco Pinto Duarte, Ivan da Rocha Pitta, Maira Galdino da Rocha Pitta

**Affiliations:** ^1^Laboratório de Imunomodulação e Novas Abordagens Terapêuticas (LINAT), Núcleo de Pesquisas em Inovação Terapêutica Suely Galdino (Nupit SG), Universidade Federal de Pernambuco (UFPE), Avenida Professor Moraes Rego, s/n, 50670-901 Recife, PE, Brazil; ^2^Serviço de Reumatologia, Hospital das Clínicas, UFPE, Avenida Professor Moraes Rego, s/n, 50670-901 Recife, PE, Brazil

## Abstract

The purpose of this paper was to evaluate the levels of IL-9 in patients with SLE and RA compared with controls and the association of IL-9 levels with clinical and laboratory parameters. IL-9 levels were assessed in 117 SLE patients, 67 RA patients, and 24 healthy controls by ELISA. Clinical and laboratory parameters were recorded. The IL-9 serum levels were significantly higher in RA patients (4,77 ± 3,618 pg/mL) and in SLE patients (12,26 ± 25,235 pg/mL) than in healthy individuals (1,22 ± 0,706 pg/mL) (*p* < 0,001). In SLE patients, there were no statistically significant associations or correlations between the levels of IL-9 and SLEDAI or other clinical and laboratorial parameters, with the exception of disease time, which showed a statistically significant negative correlation with IL-9 levels (*r* = −0,1948;  *p* = 0,0378). In RA patients, no association or statistically significant correlation was observed with disease duration, DAS28, HAQ, rheumatoid factor positivity, or erosions on radiography. These data demonstrated increased serum levels of IL-9 in SLE and RA patients, but further studies are needed to clarify the precise role of this cytokine and its potential use as therapeutic target.

## 1. Introduction

The imbalance between effector and regulatory cell populations (Treg) is of critical importance in the pathogenesis of various autoimmune disorders. According to the current paradigm, the proinflammatory axis of Th1 and Th17 cells is counter balanced by the cell populations Th2 cells and Treg [1].

Interleukin-9 (IL-9) is a member of the gamma-chain family of cytokines, first described as a member of a growing number of cytokines that have crucial roles in the development, proliferation, survival, and differentiation of multiple cell lineages of both the innate and adaptive immune systems [2].

IL-9 production was first associated with the Th2 phenotype, and many of the preliminary functions of IL-9 were tested in models of Th2-associated immunity. However, it is now known that, under specific conditions, regulatory (Tregs), Th1, and Th17 subset of T cells also express IL-9. Recently, it has been shown that IL-9-producing CD4+ T cells may represent the T helper subset Th9 cells.* In vivo* T cells produce IL-9 in both proinflammatory and anti-inflammatory environments [3].

There is some evidence to suggest that IL-9 production in Th17 cells is pathogenic during autoimmunity, particularly in type 1 diabetes, asthma, and experimental autoimmune encephalomyelitis (EAE) [4–9]. There are multiple cell types responsive to IL-9 including mast cells, T cells, antigen-presenting cells, and epithelial cells of the lung and the gut. In mast cells, IL-9 appears to promote their recruitment and expansion. IL-9 also appears to directly cause the production of TGF*β* in antigen-presenting cells. This leads to a decrease in lipopolysaccharide-induced oxidative burst and to TNF-*α* release [10, 11]. In addition to indirect effects on T cells through antigen-presenting cells, IL-9 appears to increase the suppressive effect of Treg cells and enhance the proliferation and/or accumulation of Th17 cells [6].

Recently, evidence is emerging that inappropriate regulation of Th17 cells plays a fundamental role in the development of many autoimmune diseases, including systemic lupus erythematosus (SLE) [12, 13] and rheumatoid arthritis (RA) [14]. Since IL-9 is a cytokine related to Th17 pathway, it is believed that this cytokine may be also associated with the pathogenesis of these diseases. Nevertheless, few published studies have evaluated the role of IL-9 in patients with RA [15] or SLE [16]. The purpose of this paper was to evaluate the levels of IL-9 in patients with SLE and RA compared with controls and the association of IL-9 levels with clinical and laboratory parameters.

## 2. Patients and Methods

### 2.1. Patients

Serum samples were obtained from 117 patients with SLE and 67 patients with RA who fulfilled the classification criteria of the American College of Rheumatology (ACR) [17, 18], from the Department of Rheumatology at Hospital das Clínicas da Universidade Federal de Pernambuco (UFPE). The mean age of the patients with SLE was 37,5 ± 10,41 years (range: 19 to 61 years) and for those with RA was 52,6 ± 10,15 years (range: 28 to 75 years). About 24 healthy controls were recruited in the study (all females; mean age: 35,1 ± 9,138 years, range: from 20 to 55 years). Informed consent was obtained from each patient before being included in the study. The study was approved by the ethics committee of the UFPE.

### 2.2. Collection of Clinical and Laboratory Data

The demographic, clinical, and laboratory information of the patients with SLE such as age, disease duration, gender, medication use, activity (SLEDAI) and organ damage scores (SLICC), systemic involvement and number of classification criteria, complement levels, and autoantibodies positivity were collected at the time of blood sampling and are summarized in Table 1. For the RA patients, besides demographic data, tender joint count, swollen joint count, DAS28 and CDAI values, erythrocyte sedimentation rate (ESR), and presence of rheumatoid factor (RF) were collected and are summarized in Table 1.

### 2.3. Measurement of Serum IL-9 Levels

Cytokines in the sera were assayed with ELISA kit according to the manufacturer's recommendation (eBiosciences). The lower limits of detection for ELISA IL-9 kit were 1 pg/mL.

### 2.4. Statistical Analysis

Associations of serum IL-9 levels with clinical and laboratory parameters of RA and SLE patients were analyzed by univariate comparisons using nonparametric tests (Mann-Whitney tests). *p* < 0.01 was considered as an indicator of a significant association and *p* < 0.05 as an indicator of a suggestive association. The results are shown considering the mean value. All quantitative data were plotted with Graph Pad Prism 3.02 software.

## 3. Results

**Figure 1:**
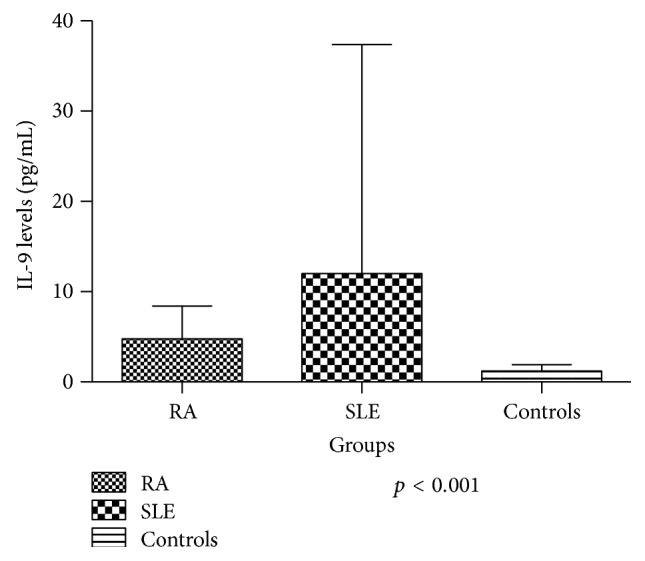
Comparison of the mean levels of IL-9 in patients with RA (*n* = 67), SLE (*n* = 117), and controls (*n* = 24).

**Figure 2:**
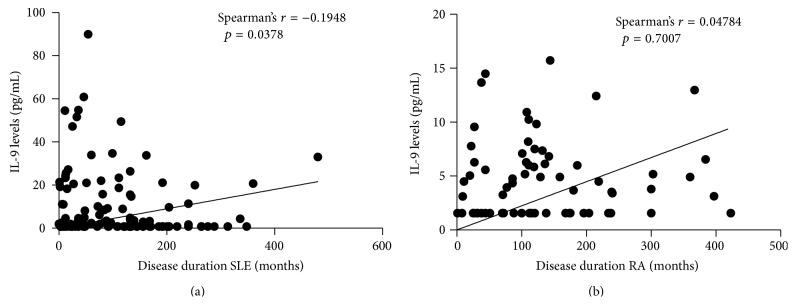
Correlation between disease duration and IL-9 levels in patients with SLE and RA.

**Figure 3:**
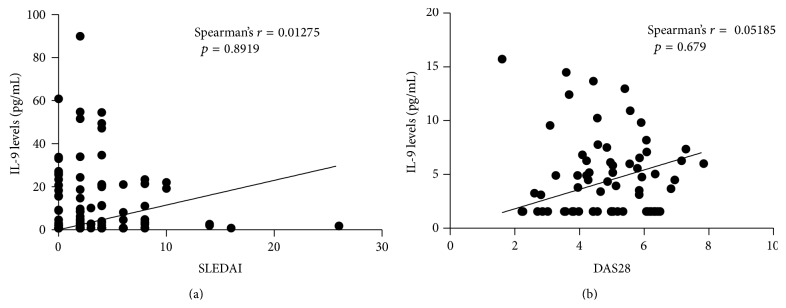
Correlation between disease activity and IL-9 levels in patients with SLE (a) and RA (b).

Serum IL-9 levels were significantly higher in RA patients (4,77 ± 3,618 pg/mL) and in SLE patients (12,26 ± 25,235 pg/mL) than in healthy individuals (1,22 ± 0,706 pg/mL) (*p* < 0,001) (Figure 1). No difference was observed between the levels of IL-9 in patients with SLE and RA, although SLE patients presented higher levels (*p* = 0,195). There were no statistically significant associations or correlations between the levels of IL-9 and SLEDAI, number of ACR criteria, organ damage, clinical manifestations, complement consumption, and ANA or anti-DNA positivity, with the exception of disease time, which showed a statistically significant negative correlation with IL-9 levels (*r* = −0,1948; *p* = 0,0378) (Figure 2). Regarding the treatment, there were no statistical differences between the various regimens used in both diseases and serum levels of IL-9. In RA patients, no association or statistically significant correlation was observed with disease duration, DAS28, HAQ, rheumatoid factor positivity, or erosions on radiography (Figure 3).

## 4. Discussion

The present study revealed that the IL-9 levels are significantly increased in SLE and RA patients compared to healthy controls; however, it has not demonstrated association with clinical or laboratory parameters in both diseases, with the exception of a negative correlation with disease duration in SLE patients.

Described for the first time in the late 1980s as a member of a growing number of cytokines that had pleiotropic functions in the immune system, it remains an understudied cytokine [19]. It has been most frequently associated with allergic inflammation [20, 21] and immunity to extracellular parasites [22, 23], although developing literature has demonstrated a role for IL-9 or IL-9-responsive cells in Th1/Th17-mediated inflammation and in T regulatory cell responses [7, 24]. The factors required for IL-9 production in T cells are being elucidated and require the integration of signals from multiple cytokines [19].

Very few studies in recent years have evaluated IL-9 in the context of rheumatic diseases such as SLE and RA. Hughes-Austin et al. [15] measured twenty-five cytokines/chemokines of first-degree relatives of RA probands, a population without RA but at increased risk for its future development. At this population, the levels of IL-9 were associated with rheumatoid factor or anti-CCP positivity. In another study, Ouyang et al. [16] showed that the plasma concentration and mRNA levels of IL-9 were significantly elevated in SLE patients compared with healthy controls and this elevation correlated with disease activity and severity. Additionally, the percentages of CD4^+^IL-9^+^ T cells and serum IL-9 levels in eight untreated active SLE patients were decreased at 1, 2, and 3 weeks after treatment with methylprednisolone, suggesting an important role of IL-9 in the pathogenesis of SLE. Burkhardt et al. [25] evaluated the association of X-chromosomal genes with RA. Two single-nucleotide polymorphisms (SNP) were associated with RA: TIMP1 that was associated with RA in general (*p* = 0.035) and IL-9 receptor (IL9R) that was associated with anti-CCP-positive RA patients (*p* = 0.037) and with male RA patients (*p* = 0.010).

Although it was originally defined as a Th2 cytokine, other T helper subsets also appear to have the potential for IL-9 production. Th17 cells, which are defined by secretion of IL-17A and IL-17F, may also secrete IL-9* in vitro* and* ex vivo* [5, 6]. Human Th17 cells can secrete IL-9, and long-term Th17 cultures have the ability to coexpress IL-17A and IL-9 [4]. In contrast, IL-23, a cytokine required for maintenance of the IL-17-secreting phenotype, has inhibitory effects on IL-9 production [6]. T regulatory cells may also produce IL-9. A study linking mast cells to peripheral tolerance demonstrated that natural Tregs (nTregs) and inducible Tregs (iTregs), both Foxp3^+^ populations, secrete IL-9 [26]. There is conflicting evidence regarding production of IL-9 from human Treg cells [4].

Recently, the Th9 cells were described as a new subset of effector T cells that develop from naive T cells in the presence of transforming growth factor *β* (TGF*β*) and interleukin-4 (IL-4). Cells cultured under these conditions are primed for the production of IL-9 and require transcription factors that include STAT6 (signal transducer and activator of transcription 6), PU.1, IRF4 (interferon response factor 4), and GATA3 [27–29]. Other recent finding is the concept that contrary to expectations based on previous results obtained* in vitro*, it is not the T cell the main cell type that expresses IL-9* in vivo* [30] but a previously unknown type of innate lymphoid cell, called the “ILC2 cell.”

The established functions of Th9 cells have been divided into type II immunity, associated with Th2 cells, and autoimmunity, associated with Th1 and Th17 cells. It has not been established if there is any immune response that is strictly dependent on Th9 cells, or if IL-9-secreting T cells function* in vivo* as a helper cell of T helper cells. A recent Chinese study demonstrated that the expression of Th9 cells (CD3^+^CD8^+^IL-9^+^) in RA patients was significantly higher than in normal controls, and this expression was positively correlated with high disease activity, elevated erythrocyte sedimentation rate (ESR), C-reactive protein (CRP), number of tender joints, the number of swollen joints, and RF [31].

Pathogenic Th17 cells mediate pannus growth, osteoclastogenesis, and synovial neoangiogenesis in RA and the imbalance between Th17 and Treg cells has been identified as a crucial event in the pathogenesis of RA [32]. Since Th9 cells through IL-9 could mediate recruitment of autoimmune Th17 cells [33], this could be a possible explanation of the role of IL-9 in the pathogenesis of RA.

Some articles described IL-9 ability to promote the survival of a large number of different cell types, including T cells, mast cells and eosinophils, neurons, tumor, and epithelial cells [34–39]. Exposure of such effector cells to IL-9 either* in vivo* as a consequence of IL-2-mediated induction of IL-9 in ILC2 cells or during* in vitro* culture might result in improved survival, that could facilitate enhanced functional responses of all T cells that express IL-9 receptor and thus mediate a diversity of different functions. This can help to explain diverse IL-9-mediated phenotypes as well as enhanced antibody production by B cells [30]. Since SLE is a disorder mediated by autoantibodies, this could be an explanation for the negative correlation found between disease duration and levels of IL-9 in our study (higher levels of IL-9 in the onset of disease), since the early event in the disease process is the breakdown of B cell tolerance and enhanced autoantibodies production [40].

In a very recent work, Yang et al. [41] demonstrated increased IL-9 levels in the spleens and kidneys of MRL/lpr mice and this was closely related to the production of antibodies against double-stranded DNA (dsDNA). IL-9 appears to promote B cell proliferation and immunoglobulin production, which could be blocked by inhibition of signal transducer and activator of transcription 3 (STAT3).* In vivo* treatment with neutralizing anti-IL-9 antibody decreased serum anti-dsDNA antibody titers and alleviated lupus nephritis in MRL/lpr mice, suggesting that IL-9 is a potential therapeutic target for SLE.

Although IL-9 was discovered decades ago, it remains one of the most enigmatic cytokines identified so far, in particular because its functional activities remain far from clear, and the literature frequently provides conflicting results on efforts to identify additional functions for IL-9. Few studies have evaluated the expression of Th9 cells or the levels of IL-9 in patients with autoimmune diseases, and it is unclear if there is an association with the pathogenesis of these diseases or if it is just an epiphenomenon [31, 42]. As IL-9 is a pleiotropic cytokine, Th9 cells might contribute to both protective immunity and immunopathological disease through a myriad of pathways. However, it is important to remember that Th9 cells are not the only source of IL-9 [43]. In allergic diseases, IL-9 production can also occur in mast cells, in part through stimuli such as histamine, IL-1, antigen-specific immunoglobulin E and antigen, and IL-9 itself, and can help to facilitate their growth and expansion.

Further work would be needed to demonstrate the extent to which mast-cell-derived IL-9 can influence immune responses in comparison to that from various T cell subsets. Despite the broad ability of IL-9 to impact multiple types of inflammation, it is not clear how it functions in the context of autoimmune diseases. Some evidence suggests that it may have pathogenic role of this cytokine in SLE and RA. Further studies, with a larger number of patients, should be performed to evaluate not only its pathogenic role but also the possible therapeutic target in these diseases.

## Figures and Tables

**Table 1:** Demographic, clinical, and laboratory data of SLE and RA patients and controls.

	SLE (*n* = 117)	%	RA (*n* = 67)	%	Controls (*n* = 24)	%
Age (years, average ± SD)	37,5 ± 10,41		52,65 ± 10,150		35,1 ± 9,138	
Disease time (months, average ± SD)	105,48 ± 93,83		135,70 ± 103,39		—	
IL-9 levels (pg/mL)	12,26 ± 25,235		4,77 ± 3,618		1,22 ± 0,706	
Gender						
Female	114	97,44	64	95,52	24	100
Male	3	2,56	3	4,48	0	0
Active nephritis	24	20,51	—	—	—	—
Rash	13	11,11	—	—	—	—
Alopecia	12	10,26	—	—	—	—
Anti-DNA positive	16	14,16	—	—	—	—
SLEDAI	3,31 ± 3,804					
SLICC	1,008 ± 1,276		—	—	—	—
Positive RF	—	—	48	72,73	—	—
Erosions^∗^	—	—	35	52,24	—	—
DAS28	—	—	4,84 ± 1,353		—	—
CDAI	—	—	20,75 ± 13,453		—	—
ESR (mm/h)	—	—	41 ± 22,000		—	—
Treatment					—	—
Corticosteroids	90	77,6	51	76,1	—	—
Antimalarials	70	60,3	13	19,4	—	—
Methotrexate	—	—	44	65,7	—	—
Leflunomide	—	—	26	38,8	—	—
Azathioprine	38	32,8	—	—	—	—
Mycophenolate mofetil	12	10,3	—	—	—	—
Biological therapy	—	—	5	7,5	—	—

^∗^Analysis for 44 patients.
